# Planning processing in ADHD with comorbid reading disabilities is worse than in ADHD: Based on Das-Naglieri Cognitive Assessment System

**DOI:** 10.3389/fped.2022.898348

**Published:** 2022-09-12

**Authors:** Zunwei Zhang, Junyan Feng, Yang Xue, Feiyong Jia, Tiantian Wang

**Affiliations:** Department of Developmental and Behavioral Pediatrics, The First Hospital of Jilin University, Changchun, China

**Keywords:** comorbidity, attention deficit hyperactivity disorder, reading disabilities, Das-Naglieri Cognitive Assessment System, planning

## Abstract

**Objective:**

To explore and compare the cognitive processing weakness of children with Attention deficit hyperactivity disorder (ADHD) and comorbid reading disabilities (RD) (ADHD+RD) and children with ADHD only using the Das-Naglieri Cognitive Assessment System (DN:CAS).

**Methods:**

Eighty-eight children with ADHD who visited the hospital for the first time from September 2021 to November 2021 and had a Full scale intelligence quotient (IQ) of ≥85 on the Wechsler Intelligence Scale for Children revised in China (C-WISC) were selected (Age: 6–12 years; Grade: 2–6). Based on comorbidity with RD and the subtypes of ADHD (e.g., Inattention dominant type, ADHD-I, Hyperactivity/Impulse dominant type, ADHD-H and Combined type, ADHD-C), these children were divided into the ADHD+RD group (*n* = 30) and ADHD group (*n* = 58) as well as the corresponding subgroups. Clinical data on gender, age, grade, IQ scores, and DN:CAS processing scores were compared between both groups/subgroups. Spearman's correlation test was used for correlation analysis of results of interest.

**Results:**

No differences in age, grade, male-to-female ratio, verbal IQ, performance IQ, and full scale IQ were observed between the ADHD+RD group and ADHD group as well as the corresponding subgroups (*P* > 0.05). Children in the ADHD-C+RD subgroup had lower scores in Planning processing of DN:CAS than those in the ADHD-C subgroup (*P* = 0.040). However, there were no significant difference between the ADHD-I+RD subgroup and ADHD-I subgroup in Planning scores of DN:CAS assessment; The grade of ADHD-C+RD and ADHD-I+RD subgroups were positively correlated with the Planning scores of DN: CAS (r = 0.599, *P* = 0.030 and r = 0.508, *P* = 0.044, respectively). The grade of ADHD-C subgroup was positively correlated with the Planning and Simultaneous processing scores of DN: CAS (r = 0.409, *P* = 0.042 and r = 0.406, *P* = 0.044, respectively).

**Conclusion:**

Our study confirmed that children of ADHD-C with comorbid RD have a more severe Planning processing weakness compared to children with ADHD-C only. Among the children of ADHD-C+RD, ADHD-I+RD and ADHD-C, such a Planning processing impairment may improve with increasing educational skills.

## Introduction

Attention deficit hyperactivity disorder (ADHD) is a neurodevelopmental disorder characterized by attention deficit, hyperactivity, impulsivity, and cognitive dysfunction. According to the different clinical manifestations, it can be divided into Inattention dominant type (ADHD-I), Hyperactivity/Impulse dominant type (ADHD-H) and Combined type (ADHD-C) (1, 2). It occurs in about 5–7% of children and 2.5% of adults (3, 4). Due to inattention is associated with poor task persistence, which can in turn result in academic underachievement. Twenty five to forty eight percent of ADHD children suffered from reading disabilities (RD) (5). RD is one of the most common neurodevelopmental disorders in childhood as well, accounting for about 80% of specific learning disabilities (SLD) (1, 4). According to the International Classification of Diseases-11 (ICD-11) and psychological definition, RD refers to the condition in which a person with adequate intelligence and equal educational opportunities shows low accuracy in word recognition, poor fluency, poor spelling ability and poor reading comprehension. Reading levels of children with RD are significantly lower than those of normal children of the same age (6). Comorbid ADHD and RD share similar genetic and neuropsychological underpinnings. For example, working memory deficits have been reported in both ADHD and RD (7); rapid automatized naming defects, a core defect of RD (8), have been documented in children with ADHD only (9, 10). However, the concurrence of ADHD and RD raises questions, as studies have suggested that the comorbidity with any two disorders can be more severe than the sum of its parts (11). When RD persist, even in moderately difficult reading tasks, children with RD may be inattentive. It is due to their inefficiency in skills needed for learning tasks, loss of sustained attention, and frustration from the arduous nature of the learning-related tasks (12). In order to further understand the adverse effects of underlying cognitive deficits of children with RD and ADHD, researchers began to focus on the neuropsychological basis, and the PASS theory (13) came into being at the end of the 20th century.

Das and Naglieri developed the PASS theory of intelligence in the 1990s based on the view of functional regions of brain operation proposed by A.R. Luria and study of cognitive psychology (13). The PASS (Planning, Attention, Simultaneous, and Successive) theory is a example of terminal cognitive processing theory. It proposes that cognition is organized in three systems. The first system is the planning system, which is the executive control system responsible for controlling and organizing behavior, selecting or constructing strategies, and monitoring performance. The second system is the attention system, which is responsible for maintaining arousal levels and alertness and ensuring that attention is focused on appropriate stimuli. The final system is the information processing system, which uses simultaneous and successive processing to encode, transform, and retain information. In simultaneous processing, the relationships between items and their units of integration into the overall information are coded. In successive processing, the information is encoded to ensure that the unique links between items are serialized in nature. Based on this theory, Naglieri and Das assembled a series of assessment tools, namely the Cognitive Assessment System (DN:CAS), which covered every component of the PASS theory (14).

There were many previous studies in children with ADHD, RD, and ADHD+RD by using DN:CAS. Most of the studies found that children with ADHD demonstrated relatively low scores on the Planning and Attention scales of the DN:CAS, but average scores on the Simultaneous and Successive scales (15–17). Some studies pointed out that children with ADHD-C had below average scores only on Planning scales (18). For RD, researchers in alphabetic countries have found that Successive processing is the core cognitive processing deficit of RD. However, Chinese is an ideographic character, it is composed of complex strokes and has no direct rules for transforming form to sound. So cognitive processing deficits in Chinese children with RD have been reported to involve both Simultaneous and Successive processing, which is different from alphabetic script (19). Japanese scholars found that ADHD children with kana dyslexia had more severe defects in simultaneous processing compared to ADHD children (20). However, the problem is also pointed out that Japanese kana characters are alphabet characters, which are different from Chinese pictographs. Therefore, the cognitive processing of the comorbidity of ADHD and Chinese RD is a field to be explored.

In our previous study, we found that the Verbal IQ, Performance IQ, and Full scale IQ scores of C-WISC of children with ADHD and comorbid SLD were significantly lower than those of children with ADHD only (21). In general, children with higher IQ are more likely and better using strategies to perform an information processing task, resulting in higher scores. Hence, studies have shown that children with greater intelligence have better compensatory skills to help them overcome RD. Therefore, it becomes very interesting to study the cognitive processing defects of RD with high intelligence level. To solve this proposition, the Full scale IQ score of all children selected in this study was ≥85 points, which was as close as possible to the 90-point cut-off at the general level. In addition, due to the cognitive processing among different subtypes of ADHD were not the same in previous studies. Therefore, we defined ADHD subtype to ensure homogenization as much as possible. This study sought to explore and compare the cognitive processing weakness of children with ADHD+RD and those with ADHD only using the DN:CAS assessment to find a more suitable cognitive intervention training or approach.

## Methods

### Participants

A total of 58 children with ADHD and 30 children with ADHD+RD were recruited in this study. All the children were enrolled from the Developmental Behavioral Pediatrics Clinic of the First Hospital of Jilin University from September 2021 to November 2021. ADHD was diagnosed according to the Diagnostic and Statistical Manual of Mental Disorders, Fifth Edition (DSM-5) criteria. Detailed clinical information should be collected for the diagnosis of ADHD, and the Vanderbilt ADHD scales (22, 23) (including parent version and teacher version) were used to fill in the behavior of the child in the last 6 months. The parent's version of the scale was completed by the child's father or mother immediately. The teacher's version of the scale was completed at the appropriate time after the child's parents have completed their version and passed it on to the head teacher. Thereafter, the child and his/her guardians returned to the clinic for final diagnosis by the same senior specialist. All study subjects were between the ages of 6 and 12 years, and the grades ranged from grade 2 to grade 6. C-WISC assessment was conducted by the professional evaluators (24). Children with a Full scale IQ ≥ 85 were selected. Children with organic diseases of the nervous system, intellectual disability, epilepsy, autism spectrum disorders and other severe neurodevelopmental disorders, major physical diseases, and taking any medication for ADHD, or being engaged in psychotherapy to manage ADHD symptoms were excluded from the study. The assessment of RD was performed on children diagnosed with ADHD. First, children with ADHD whose reading performance (reading fluency and reading comprehension) and reading achievements were within the bottom 10% of all children in the same grade and scored <65 on the Learning Disability Screening Scale (PRS) (25) were selected. Thereafter, the parents filled the Dyslexia Checklist for Chinese Children (DCCC) (26), and RD was considered if the DCCC score was higher than the cut-off value. Participants who met criteria for ADHD without reading disability were placed into one group. Participants who met criteria for ADHD+RD were placed into another group. Each group was then divided into one of the three subgroups according to the ADHD subtypes. After the guardian provided informed consent, the DN:CAS processing assessment was performed when the child was in good condition. All the measures of the participants in our study were generally administered within 3 days. Clinical data, including sex, age, grade, IQ scores, and DN:CAS processing scores, were compared between groups/subgroups. Additionally, correlation analysis on the results of interest was performed.

### Instrument

#### Vanderbilt ADHD rating scale

The VARS was developed by Mark Wolraich of the University of Oklahoma according to the diagnostic criteria for ADHD in the DSM-IV. The toolkit is suitable for children aged 6–12 years. It consists of two subscales: Vanderbilt ADHD Diagnostic Parent Rating Scale (VADPRS) and Vanderbilt ADHD Diagnostic Teacher Rating Scale (VADTRS). Both subscales include behavior and performance assessment. For VADPRS, the section on behavior includes 47 items, which were divided into attention deficit (items 1–9), hyperactivity/impulsivity (items 10–18), oppositional defiance (items 19–26), conduct disorder (items 27–40), and anxiety/depression (items 41–47). For the VADTRS, the section on behavior includes 35 items, which are divided into attention deficit (items 1–9), hyperactivity/impulsivity (items 10–18), oppositional defiance (items 19–28), and anxiety/depression (items 29–35). Each item was graded at four levels: never (0), occasionally (1), often (2), and always (3). The performance section of both VADPRS and VADTRS contains eight items, which can quickly assess children's learning ability and interpersonal communication. Each item is rated as excellent (1), above average (2), average (3), somewhat problematic (4), or problematic (5). In 1998 and 2003, Professor Wolraich and other scholars conducted psychometric analysis on VADTRS and VADPRS, which showed that the scale had good reliability and validity (22, 23).

#### Wechsler intelligence scale for children revised in China

WISC (24) was used to evaluate the children's intellectual achievement. This C-WISC assessment tool is suitable for school-age children aged 6–16 years, which was developed by Gong Yaoxian et al. in 1994 based on the Revised Wechsler Intelligence Scale for Children (WISC-R). It consisted of Full scale IQ, Verbal IQ, and Performance IQ. Verbal IQ consisted of five factors, including information, similarities, arithmetic, vocabulary, and recitation; Performance IQ consisted of five factors, including picture completion, picture arrangement, block design, object assembly, and coding. The original scores of the full scale and subscales were converted into standard scores according to the norm. The reliability and validity of C-WISC in the Chinese-speaking setting were 0.86 and 0.75, respectively (24). Children in this study were assessed by qualified and standardized trained professionals.

#### The pupil rating scale–revised screening for learning disability

PRS is widely used to screen learning disabilities in China. It comprises the following five functional areas of verbal and non-verbal types: auditory comprehension and memory, language, time and orientation judgment, movement, and social behavior. The questionnaire has good reliability (retest correlation coefficients over 0.80) and fair validity (criterion validity correlation coefficients from 0.53 to 0.63) (25). The headteacher completed the questionnaire according to the students' performance in school.

#### Dyslexia checklist for Chinese children

DCCC (26) applies to children in grades 2–6. The eight dimensions, including the deficit of vocabulary comprehension, visual deficit of word recognition, auditory deficit of word recognition, deficit of spelling, deficit of written expression and attention, deficit of oral language, and bad reading habits, comprised 55 items. The score of each item ranged from 1 to 5 (1, never; 2, seldom; 3, sometimes; 4, often; 5, always). The highest score represented the worst reading ability. The original score of each item was summed and converted to a T score [T = 50 + 10 (X-M)/SD]. A T-score > 70 was considered abnormal. The reliability and validity were 0.974 and 0.930, respectively.

#### DN:CAS Chinese version

DN:CAS is an intelligence test that operationalizes the PASS theory. The assessment system was suitable for individuals aged 5–17 years and included four subscales: Planning, Attention, Simultaneous processing, and Successive processing.

Planning is involved in the cognitive process of executive function (i.e., decision, selection, and effective use of strategies). The plan is modified as needed to stay consistent with the original goal. Planning assessment involves matching numbers and planned codes and planned connections.

Attention processing includes expressive attention, number detection, and receptive attention. It involves focusing on cognitive activities, detecting specific stimuli, and inhibiting responses to distractive stimuli.

Simultaneous processing includes non-verbal matrices, verbal spatial relations, and figure memory. This assessment requires the child to perceive the relationship between the components of the item and to integrate the separate elements into a complete pattern or concept of interrelation using verbal or non-verbal content.

Successive processing assessment includes three tasks—word series, sentence repetition, and sentence questioning—which require individuals to understand and grasp information presented in a specific order.

In this study, the Chinese version of the DN:CAS (27), which had good reliability, criterion-related validity, and construct validity, was adopted.

### Statistical analysis

SPSS 22.0 statistical software (SPSS for Windows, SPSS Inc., Chicago, IL, USA) was used for all data analyses. Student's *t*-test was used for comparison between groups. For categorical data, χ^2^ test was used for analyzing the difference between groups. The correlations among the DN:CAS scores and grade were detected using the Spearman's correlation test. *P*-value <0.05 was considered statistically significant.

## Results

Detailed demographic data, IQ scores of C-WISC and the proportion of each ADHD subtype are available in Table 1.

**Table 1:** Basic information of the ADHD+RD and ADHD groups.

**Groups**	**ADHD+ RD** **(*n* = 30)**	**ADHD** **(*n* = 58)**	***P-*value**
Age (years)	8.55 ± 1.38	8.65 ± 1.67	0.781
Gender (male:female)	25:5	44:14	0.419
Grade	2.90 ± 1.16	3.28 ± 1.57	0.249
Verbal IQ	97.63 ± 7.90	98.03 ± 8.60	0.832
Performance IQ	96.77 ± 9.03	100.29 ± 8.60	0.077
Full scale IQ	96.90 ± 7.63	99.16 ± 7.35	0.181
ADHD subtype			
Combined	13 (43.3%)	25 (43.1%)	0.992
Inattentive	16 (53.3%)	31 (53.4%)	0.984
Hyperactive/impulsive	1 (3.3%)	2 (3.4%)	0.978

Due to the small number of ADHD-H subtypes (ADHD-H+RD, *n* = 1; ADHD-H, *n* = 2) included in this study, it was not statistically analyzed.

### Comparison of demographic data and IQ scores between the ADHD-C+RD subgroup and ADHD-C subgroup

There were no differences in age, grade, the ratio of male to female, Verbal IQ, Performance IQ and Full scale IQ were observed between the ADHD-C+RD subgroup and ADHD-C subgroup (*P* > 0.05) (Table 2A).

**Table 2A:** Basic information of the ADHD-C+RD and ADHD-C subgroups.

**Groups**	**ADHD-C+ RD** **(*n* = 13)**	**ADHD-C** **(*n* = 25)**	***P-*value**
Age (years)	8.55 ± 1.28	8.60 ± 1.75	0.928
Gender (male:female)	11:2	21:4	0.961
Grade	2.85 ± 1.21	3.28 ± 1.67	0.414
Verbal IQ	96.38 ± 6.87	97.88 ± 9.19	0.610
Information	9.54 ± 2.60	9.32 ± 2.56	0.805
Similarities	10.69 ± 2.43	10.52 ± 2.34	0.828
Arithmetic	7.92 ± 2.57	10.28 ± 2.75	0.015*
Vocabulary	9.69 ± 3.17	8.68 ± 2.02	0.237
Digit span	9.23 ± 2.05	9.28 ± 2.30	0.949
Performance IQ	96.62 ± 10.04	98.84 ± 7.94	0.459
Picture completion	8.62 ± 2.63	9.56 ± 1.76	0.195
Picture arrangement	9.23 ± 2.49	8.60 ± 2.65	0.482
Block design	10.85 ± 1.73	11.08 ± 2.52	0.766
Object assembly	9.69 ± 2.90	10.64 ± 1.71	0.211
Coding	9.31 ± 3.23	9.60 ± 2.45	0.756
Full scale IQ	96.15 ± 7.22	98.24 ± 7.36	0.410

There is no significant difference in age, grade (student's *t*-test)and gender distribution (χ^2^ test) between different groups, and there are no significant differences in Verbal IQ, Performance IQ and Full scale IQ between the two groups. The data are shown as mean ± SD or ratio. IQ, intelligence quotient; ADHD-C, attention deficit hyperactivity disorder combined type; RD, reading disabilities. *means *P* < 0.05.

### The DN:CAS assessment of the ADHD-C+RD subgroup and ADHD-C subgroup

We found that both children with ADHD-C+RD and ADHD-C had a relatively low scores on the Planning and Attention scales of the DN:CAS, but average scores on the Simultaneous and Successive scales. However, children in the ADHD-C+RD subgroup had significantly lower scores in Planning assessment of DN:CAS compared to children in the ADHD-C subgroup (*P* = 0.040) (Table 2B).

**Table 2B:** DN: CAS full scale and subscale scores for ADHD-C+RD subgroup and ADHD-C subgroup.

**Groups**	**ADHD-C+ RD** **(*n* = 13)**	**ADHD-C** **(*n* = 25)**	***P-*value**
Planning	75.00 ± 10.58	83.60 ± 12.39	0.040*
Matching numbers	7.15 ± 2.54	8.04 ± 3.03	0.374
Planned codes	5.62 ± 1.66	7.16 ± 1.63	0.009**
Planned connections	5.23 ± 2.28	6.96 ± 2.85	0.067
Simultaneous processing	111.54 ± 10.49	112.00 ± 10.30	0.897
Non-verbal matrices	12.31 ± 2.46	13.08 ± 2.47	0.365
Verbal-spatial relations	12.00 ± 3.51	10.92 ± 3.46	0.370
Figure memory	11.38 ± 3.45	11.96 ± 3.01	0.598
Attention	85.92 ± 12.96	88.16 ± 9.97	0.558
Expressive attention	8.23 ± 3.03	9.36 ± 2.96	0.275
Number detection	6.92 ± 2.60	7.68 ± 2.38	0.373
Receptive attention	7.92 ± 3.33	7.08 ± 2.38	0.373
Successive processing	106.31 ± 7.87	111.68 ± 9.93	0.100
Word series	15.54 ± 3.02	16.52 ± 2.82	0.326
Sentence repetition	7.77 ± 1.59	8.12 ± 1.99	0.585
Sentence questions	9.92 ± 2.78	11.40 ± 2.42	0.098
Full scale	92.62 ± 10.26	98.36 ± 9.11	0.086

Comparison of DN: CAS full scale and subscale scores between ADHD-C+RD subgroup and ADHD-C subgroup were done by student's *t-*test, ^*^means *P* < 0.05, ^**^means *P* < 0.01.The data are shown as mean ± SD.

### Comparison of demographic data and IQ scores between the ADHD-I+RD subgroup and ADHD-I subgroup

There were no differences in age, grade, the ratio of male to female, Verbal IQ, Performance IQ and Full scale IQ were observed between the ADHD-I+RD subgroup and ADHD-I subgroup (*P* > 0.05) (Table 3A).

**Table 3A:** Basic information of the ADHD-I+RD and ADHD-I subgroups.

**Groups**	**ADHD-I+RD** **(*n* = 16)**	**ADHD-I** **(*n* = 31)**	***P-*value**
Age (years)	8.57 ± 1.56	8.80 ± 1.61	0.651
Gender (male:female)	13:3	21:10	0.327
Grade	3.00 ± 1.16	3.35 ± 1.52	0.861
Verbal IQ	98.50 ± 8.96	97.77 ± 8.37	0.964
Information	9.63 ± 2.83	9.26 ± 2.64	0.930
Similarities	10.75 ± 2.38	10.84 ± 2.06	0.581
Arithmetic	9.38 ± 1.89	9.23 ± 2.28	0.122
Vocabulary	10.19 ± 2.66	9.65 ± 2.50	0.124
Digit span	8.88 ± 2.31	9.35 ± 2.37	0.906
Performance IQ	97.13 ± 8.72	101.06 ± 9.07	0.339
Picture completion	9.63 ± 1.67	9.71 ± 1.94	0.766
Picture arrangement	9.06 ± 2.41	9.00 ± 2.45	0.560
Block design	10.31 ± 2.77	11.97 ± 2.82	0.225
Object assembly	9.81± 1.87	9.94 ± 2.67	0.258
Coding	9.13 ± 2.34	10.16 ± 2.40	0.392
Full scale IQ	97.56 ± 8.35	99.45 ± 7.32	0.542

There is no significant difference in age, grade (student's *t*-test)and gender distribution (χ^2^ test) between different groups, and there are no significant differences in Verbal IQ, Performance IQ and Full scale IQ between the two groups. The data are shown as mean ± SD or ratio. IQ, intelligence quotient; ADHD-I, attention deficit hyperactivity disorder inattention type; RD, reading disabilities.

### The DN:CAS assessment of the ADHD-I+RD subgroup and ADHD-I subgroup

We found that both children with ADHD-I+RD and ADHD-I had a relatively low scores on the Planning and Attention scales of the DN:CAS, but average scores on the Simultaneous and Successive scales. And there were no differences of DN:CAS scores between ADHD-I+RD subgroup and ADHD-I subgroup (*P* > 0.05) (Table 3B).

**Table 3B:** DN: CAS full scale and subscale scores for ADHD-I+RD subgroup and ADHD-I subgroup.

**Groups**	**ADHD-I+ RD** **(*n* = 16)**	**ADHD-I** **(*n* = 31)**	***P-*value**
Planning	80.81 ± 12.56	83.65 ± 9.96	0.403
Matching numbers	8.50 ± 4.00	8.74 ± 2.86	0.812
Planned codes	6.38 ± 1.54	6.90 ± 1.66	0.296
Planned connections	5.81 ± 2.56	6.58 ± 2.08	0.250
Simultaneous processing	111.44 ± 13.92	115.68 ± 13.38	0.315
Non-verbal matrices	12.38 ± 2.60	13.29 ± 3.12	0.320
Verbal-spatial relations	10.69 ± 3.05	11.77 ± 2.06	0.154
Figure memory	11.56 ± 3.41	12.74 ± 3.40	0.266
Attention	91.38 ± 9.52	87.68 ± 12.56	0.307
Expressive attention	9.13 ± 2.68	8.55 ± 3.04	0.525
Number detection	7.06 ± 2.57	7.45 ± 1.80	0.548
Receptive attention	8.88 ± 4.09	7.94 ± 3.83	0.438
Successive processing	109.00 ± 9.71	109.32 ± 8.91	0.910
Word series	16.06 ± 2.91	17.03 ± 2.07	0.193
Sentence repetition	7.50 ± 2.10	8.10 ± 1.60	0.282
Sentence questions	9.88 ± 3.56	9.71 ± 2.78	0.862
Full scale	97.56 ± 9.75	98.71 ± 10.74	0.722

Comparison of DN: CAS full scale and subscale scores between ADHD-I+RD subgroup and ADHD-I subgroup were done by student's *t-*test. The data are shown as mean ± SD.

### Correlations between grade and the Das-Naglieri Cognitive Assessment System sub-scale scores

The grades of ADHD-C+RD and ADHD-I+RD subgroups were positively correlated with the Planning scores of DN: CAS (r = 0.599, *P* = 0.030 and r = 0.508, *P* = 0.044, respectively). The grade of ADHD-C group was positively correlated with the Planning and Simultaneous processing scores of DN: CAS (r = 0.409, *P* = 0.042 and r = 0.406, *P* = 0.044, respectively) (Table 4).

**Table 4:** Correlations between grade and the Das-Naglieri Cognitive Assessment System sub-scale scores.

	**Planning**	**Simultaneous**	**Attention**	**Successive**	**Full scale**
Grade of ADHD-C+RD	0.599*	0.160	0.394	0.167	0.469
Grade of ADHD-C	0.409*	0.406*	−0.146	0.131	0.344
Grade of ADHD-I+RD	0.508*	0.306	−0.228	0.253	0.397
Grade of ADHD-I	0.061	−0.249	−0.128	−0.323	−0.195

The correlations were analyzed by Spearman's correlation. ^*^means *P* < 0.05.

## Discussion

Eighty eight ADHD children with equal or <85 points of Full scale IQ according to the C-WISC were enrolled in our study. They were divided into six subgroups which are ADHD-C+RD, ADHD-C, ADHD-I+RD, ADHD-I, ADHD-H+RD and ADHD-H according to the presence of comorbid RD and the subtypes of ADHD. Due to the small number of ADHD-H+RD (*n* = 1) and ADHD-H (*n* = 2) subgroups, the three children were not included in the statistics. No statistical differences in age, sex ratio, grade (years of schooling), Verbal IQ, Performance IQ and Full scale IQ scores were observed in all comparison. DN:CAS assessment was used to investigate the cognitive processing characteristics in ADHD+RD and ADHD children, and the differences between groups. The results show that ADHD-C+RD had lower scores in Planning processing when compared to ADHD-C. However, we found no significant differences between ADHD-I+RD and ADHD-I in all subscales of DN:CAS. Interestingly, years of schooling of children in ADHD-C+RD and ADHD-I+RD were positively correlated with their Planning processing of DN:CAS. In addition, the years of schooling were positively correlated with Planning and Simultaneous processing scores of DN: CAS in children with ADHD-C. Such correlations were not found in the ADHD-I.

In this study, it was showed that all the ADHD children, with or without RD, had relatively low DN:CAS scores in the Planning and Attention scales, and average scores in the Simultaneous and Successive scales. These results are consistent with the previous studies (15–17). In the other hand, we discovered that the DN: CAS Planning score of ADHD-C+RD is further declined compared to ADHD-C. Planning is a set of decisions or strategies that individuals use and modify to solve problems and achieve goals (28). In fact, increasing evidences suggest that executive dysfunction has been identified in children with RD (29, 30). In addition, children with RD have been reported to show higher cognitive impulsivity (31). Purvis and Tannock (30) found that children with ADHD+RD had higher levels of cognitive impulsivity than children with ADHD. Another study believed that the neuropsychological background of the co-occurrence of RD and ADHD overlapped, which could be understood as the coexistence of short-term phonetic memory deficits and central executive function deficits (32). Planning in the DN:CAS assessment mainly tests the executive function of the cognitive processing. The cognitive impulsiveness and central executive function defects mentioned in these studies have been further confirmed in our study. However, the ability of Planning processing in children with ADHD-I+RD show no significant difference compared to ADHD-I children. The reason could due to the small sample size, or the fact that the organizing and planning ability of ADHD-I children are not seriously impaired. These children still have some self-regulating ability and adaptability.

In addition, we found that the cognitive impairments of children with ADHD comorbid Chinese RD were different from those of children with ADHD comordid alphabetic characters RD. The children with ADHD comorbid alphabetic characters RD were reported to have poorer Simultaneous processing performance. Chinese is different from alphabetic characters. In the process of reading Chinese characters, there are three decoding steps. The first step is to activate the graphic recognition, then comes phonological activation, and the last step is the semantic priming. The completion of this reading process may need more Planning processing to better finish the conversion of graphic, phonological, and semantic of Chinese characters. Hence, Planning deficits could be presented in these Chinese ADHD children who had RD.

Furthermore, we selected the grade (years of schooling) as the point of interest and conducted correlation analysis with the cognitive processing of each subgroup. The Planning scores of ADHD-C+RD, ADHD-I+RD and ADHD-C children were positively correlated with their grade, whereas no such correlation was present in the ADHD-I subgroup. Some studies found that the relationship between cognitive processing and reading increased with age. However, no unified answer explains why the relationship is increased. One possible hypothesis is that the skills required for reading become more complex with age, increasing the involvement of the four PASS processes (33, 34). We believe that with the increase in children's years of schooling, the difficulty of reading and skills needed to master become more difficult. In addition, children have more opportunities to improve their reading skills accordingly. In this case, planning processing, one of the general cognitive processing skills, needs to adapt to this change, and may be promoted. ADHD-C children may directly benefit from the management of education. The results are encouraging, suggesting that appropriate strategies for targeted instruction or intervention in children's learning to read may substantially improve cognitive processing, which could benefit reading performance. Since the Planning processing is not the main defect of ADHD-I, it is easy to understand that there is no observed correlation with years of schooling. These results also tell us from another perspective that different subtypes of ADHD and whether combined with RD are different, and attention should be paid not to confuse them into a same disease in clinical work.

## Limitations and further directions

This study had several limitations. First, since the Chinese language has pictographic characters and the English language has phonetic characters, we could not determine whether the cognitive processing characteristics of Chinese children with RD are similar to those of English children with RD. This limitation may weaken the comparability between the results of this study and previous studies to some extent. Second, due to the lack of a completely healthy control group, we could not determine the cognitive gap between children with ADHD+RD and their healthy peers. Therefore, our results can only be limited to the ADHD group. Third, this study excluded children with a Full scale IQ <85 on the C-WISC. Although the diagnoses of both RD and ADHD require adequate intelligence, Full scale IQ between 70 and 85 points is allowed. Moreover, quite a number of children with RD have a Full scale IQ <85. The results of this study could not represent the cognitive processing ability of all children with ADHD+RD. Subsequently, we will investigate whether children with lower Wechsler intelligence have different cognitive processing characteristics.

## Conclusion

Our study confirmed that children of ADHD-C+RD have more severe Planning processing weakness compared to children with ADHD-C. In contrast, there were no obvious differences between ADHD-I+RD and ADHD-I. But such impairment may improve with increasing educational skills among the children of ADHD-C+RD, ADHD-I+RD, and ADHD-C. And this conjecture should be identified and assessed with stronger evidence.

## Data availability statement

The raw data supporting the conclusions of this article will be made available by the authors, without undue reservation.

## Ethics statement

The studies involving human participants were reviewed and approved by Ethical Committee of Jilin University First Hospital. Written informed consent to participate in this study was provided by the participants' legal guardian/next of kin. Written informed consent was obtained from the individual(s), and minor(s)' legal guardian/next of kin, for the publication of any potentially identifiable images or data included in this article.

## Author contributions

ZZ acquired the clinical data, reviewed the literature, and drafted the article. TW designed the study, supervised the initial drafting, and critically revised the article. JF, YX, and FJ analyzed the clinical data and critically revised the article. All authors approved the final version of the article.

## Funding

This work was supported by the National Natural Science Foundation of China (Grant Number: 81973054), Key Scientific and Technological Projects of Guangdong Province (Grant Number: 2018B030335001), and the Joint Fund Bethune Medical Special Project of Jilin Province (Grant Number: 20200201507JC).

## Conflict of interest

The authors declare that the research was conducted in the absence of any commercial or financial relationships that could be construed as a potential conflict of interest.

## Publisher's note

All claims expressed in this article are solely those of the authors and do not necessarily represent those of their affiliated organizations, or those of the publisher, the editors and the reviewers. Any product that may be evaluated in this article, or claim that may be made by its manufacturer, is not guaranteed or endorsed by the publisher.
